# Prevalence and Risks Factors of Prehypertension in Africa: A Systematic Review

**DOI:** 10.5334/aogh.2769

**Published:** 2022-03-01

**Authors:** Koussoh Simone Malik, Kassi Anicet Adoubi, Jérôme Kouame, Madikiny Coulibaly, Marie-Laure Tiade, Serge Oga, Michèle Ake, Odile Ake, Luc Kouadio

**Affiliations:** 1 National Institute of Public Health, Abidjan, CI; 2 Alassane Ouattara University, Bouaké, CI; 3 Department of Public Health, Hydrology and Toxicology, Faculty of Pharmaceutical and Biological Sciences, University Felix Houphouet Boigny, Abidjan, CI

## Abstract

**Background:**

Hypertension is one of the major factors for high mortality of adults in Africa. However, complications occur at lower values than those previously classified as hypertension. Thus, prehypertension is considered as a new category of hypertension and a major risk factor for developing clinical hypertension relative to those with normotension, it has been linked with increased future risk of hypertension as well as cardiovascular diseases.

**Objectives:**

The objective of this review was to determine prevalence of prehypertension and describe the associated factors of prehypertension in Africa during the past 10 years.

**Methods:**

We did a systematic review using the databases PubMed/Medline, and search engine google scholar. We selected sources of publications and conducted an analysis of articles. Keywords in English were: prehypertension, high normal blood pressure, high blood pressure, elevated blood pressure, Africa. Keywords in french were: préhypertension artérielle, préhypertension, pression artérielle normale haute, pression artérielle normale, Afrique.

**Mesh terms were:**

Prehypertension, Africa.

**Results:**

Twenty-seven articles were selected. Prevalence of prehypertension ranged from 2.5% to 34% in children and adolescents. In adults, prevalence varied from 32.9% to 56.8%. Several factors were associated with prehypertension in Africa. These factors included: age; sex; lifestyle such as smoking, alcohol consumption, low physical activity, overweight and obesity. There were also cardiometabolic factors and few others factors which were associated with prehypertension.

**Conclusion:**

This review allowed us to observe that the prevalence of prehypertension was variable according to age of the population and prehypertension is associated with several factors.

## Introduction

It is estimated that more than 1.5 billion people suffer from hypertension [1,2]. The global prevalence of hypertension is expected to increase from 26% in 2000 to 29.2% in 2025 [6], and is among the leading contributors to the global burden of disease and premature death, accounting for approximately 9.4 million deaths annually [3,4]. Hypertension is one of the major factors for high mortality of adults in Africa. Hypertension is a modifiable traditional risk factors of cardiovascular diseases and has attracted a lot of attention due to its high morbidity and mortality, however complications occur at lower values than those previously classified as hypertension [5]. Indeed, the positive relationship between blood pressure and cardiovascular risk has also been demonstrated not only in patients with hypertension, but also in individuals with high normal blood pressure [6]. Thus, the Seventh Joint National Committee on Prevention, Detection, Evaluation and Treatment of High Blood Pressure (JNC VII) focused on increasing the risk associated with high blood pressure and defined the concept of prehypertension in 2003 [7].

Prehypertension has been defined as a systolic blood pressure between 120 mmHg and 139 mmHg and/or a diastolic blood pressure between 80 and 89 mmHg [7]. In children “prehypertension” was defined as systolic blood pressure (SBP) and/or diastolic blood pressure (DBP) ≥ 90th percentile and < 95th percentile (on the basis of age, sex, and height tables). For adolescents, “prehypertension” was defined as blood pressure (BP) ≥ 120/80 mm Hg and < 95th percentile, or BP ≥ 90th and < 95th percentile [8].

Prehypertension is considered as a new category of hypertension and a major risk factor for developing clinical hypertension relative to those with normotension, it has been linked with increased future risk of hypertension as well as cardiovascular diseases [6]. A systematic review on prehypertension was carried out in Africa, but it only concerned children and adolescents [9]. Some systematic reviews have focused on hypertension in Africa, either in older adults [10] or in children and adolescents [11]. Most of these reviews have been coupled with meta-analysis. Our research concerned children, adolescents and adults. Thus, the objective of this review is to determine prevalence of prehypertension and describe the associated factors of prehypertension in Africa during the past 10 years.

## Methods

### Study design

This systematic review was conducted following PRISMA (Preferred Reporting Items for Systematic Reviews and Meta-Analyses) guidelines [12]. Prehypertension was defined as:

- Systolic BP 120–139 mmHg and diastolic BP 80–89 mmHg according to the JNC VII report [7] in adults.
- In children “prehypertension” was defined as systolic blood pressure (SBP) and/or diastolic blood pressure (DBP) ≥ 90th percentile and < 95th percentile (on the basis of age, sex, and height tables). For adolescents, “prehypertension” was defined as blood pressure (BP) ≥ 120/80 mm Hg and < 95th percentile, or BP ≥ 90th and < 95th percentile [8].

The method used in this study was a survey of the literature for relevant studies on the prevalence of prehypertension and associated factors in Africa from January, 2011 to November, 2021.

### Literature review: database and search engine

We did a comprehensive literature review using the databases PubMed/Medline, and search engine google scholar. We selected the sources of the publications and conducted an analysis of the articles in order to keep the most relevant ones concerning our problematic.

#### Selection criteria

Selection was made on the basis of reading the titles and the abstracts, then by reading the body of the article.

##### Inclusion criteria

Inclusion criteria were constituted by:

- The types of articles (Original articles, book chapter).
- *The language (English and French)*.
- Location (Africa).
- The date of publication (the last ten years).

We included papers reporting prehypertension among children and dolescents or adults and papers in wich blood pressure measurement was performed according to guideline. We excluded studies using a single measurement of blood pressure. We also excluded articles in which the conditions for measuring blood pressure were not specified and articles reporting systolic or diastolic prehypertension separately.

##### Non-inclusion criteria

Letters to editor, editorials, theses and reports were not included in this review.

#### Mesh terms and keywords

For search in the database and the search engine, we used the following Mesh terms and keywords in English and French:

- The Mesh terms were: prehypertension, Africa
- The keywords in English were: prehypertension, high normal blood pressure, High blood pressure, elevated blood pressure, Africa
- The keywords in French were: préhypertension artérielle, préhypertension, pression artérielle normale haute, Afrique.

#### Research strategy

The various keywords and Mesh Terms have been combined using « AND » in English and « ET » in French. The equations that allowed us to do the google scholar search were:

- « préhypertension artérielle ET Afrique »
- « pression artérielle normale haute ET Afrique »
- « pression artérielle normale ET Afrique »

those used for the PubMed search were:

- « prehypertension AND Africa »
- « high normal blood presure AND Africa »
- « high blood pressure AND Africa »
- « elevated blood pressure AND Africa »
- « ([Prehypertension AND Africa] AND [High blood pressure AND Africa]) AND (elevated blood pressure AND Africa) »

#### Articles selection

The selection of articles was done in two steps. The first step consisted in the analysis by reading the titles and abstracts of the articles; then, the second step allowed us to read the body of the articles and select those corresponding to the inclusion criteria. Two investigators (MKS and KJ) independently extracted relevant data from individual studies using a preconceived data extraction form.

Information extracted included first author’s name, year of publication, population age, prevalence of prehypertension, sample size, risks factors and study country. Disagreements between authors were reconciled with another author (CM) through discussion and consensus.

**Figure 1:**
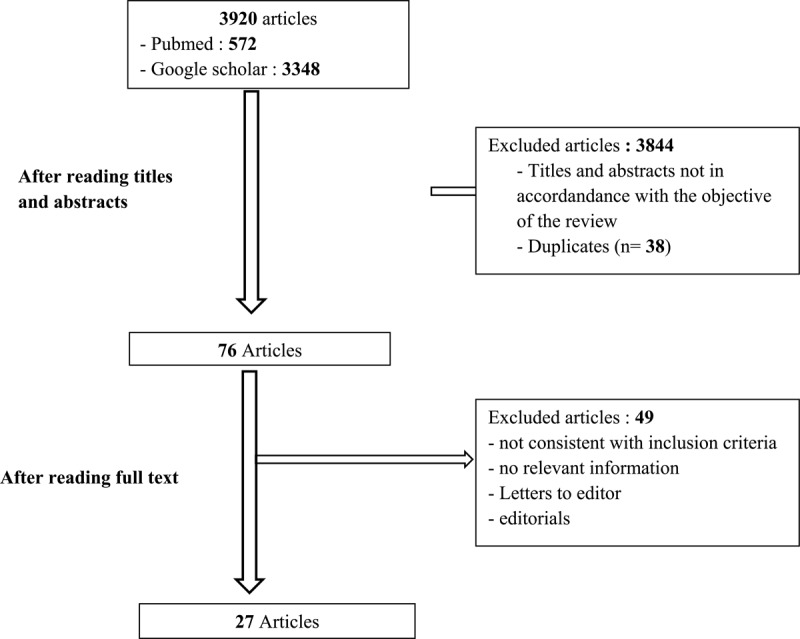
Flow diagram of the study selection process.

Figure 1 describes the selection procedure and the reasons for excluding articles. These 27 articles come from 12 countries.

## Results

A total of 3920 articles were identified by combining keywords when searching the Medline/PubMed database and the google scholar search engine. We selected 572 with the PubMed database and 3348 with the google scholar search engine. After reading the titles and abstracts, 76 articles were selected. Finally, 27 articles were selected for this review.

Table 1 shows some characteristics of the articles which are authors, population age, prevalence of prehypertension (preHTN), sample size, risks factors and study country.

**Table 1:** Articles characteristics.


AUTHORS	POPULATION AGE (YEARS)	PREVALENCE OF PREHTN	SAMPLE SIZE	RISKS FACTORS	STUDY COUNTRY

Redjala et al. 2021 [13]	6–18	10.0¨%	3562	– overweight/obesity – >2 hours/day spent watching TV, internet and electronic games – parental hypertension or diabetes – shorter gestational age (33 – 36 weeks) – early birth, – reduced birth weight, – shorter breastfeeding	Algiers

Ongosi et al. 2020 [14]	25–64	Male: 49.0% Female: 43.7%	593	– men – overweight/obesity – Low physical activity, – Low fruit and vegetable intake	Kenya

Sungwa et al. 2020 [15]	6–16	9.6%	742	– women – overweight/obesity – age > 10 years – eating fried food – drinking sugar soft drinks – not eating fruits	Tanzania

Umuerri et Aiwuyo 2020 [16]	≥18	42.5%	852	– age – body mass index – place of residence – level of education – employment status – fruit intake	Nigeria

Katamba et al. 2020 [17]	12–19	7.1%	616	Not evaluated	Uganda

Owiredu et al. 2019 [18]	≥25	49.0%	204	– having lower level of education – not practicing at least 30 min daily walks – not exercising routinely – alcohol consumption	Ghana

Nsanya et al. 2019 [19]	12–24	29%	1596	– men – obesity – age > 20 years – not eating fruits and vegetables	Tanzania and Uganda

Muhihi et al. 2018 [20]	6–17	4.4%	446	– overweight/obesity – age > 10	Tanzania

Osei-Yeboah et al. 2018 [21]	22–59	52.68%	112	Not evaluated	Ghana

Bhimma et al. 2018 [22]	16.2–21.7	29.7%	575	– overweight/obesity – male gender	South Africa

Msemo et al. 2018 [23]	18–40	37.2%	1247	– increasing age, – obesity – haemoglobin levels	Tanzania

Ezeudu et al. 2018 [24]	10–19	5.0%	984	– overweight/obesity – public school	Nigeria

Okpokowuruk et al. 2017 [25]	3–17	2.5%	200	– age – BMI – waist circumference	Nigeria

Mosha et al. 2017 [26]	≥15	36.2%	9678	– level of education – rural areas – overweight/obesity – Alcohol and tobacco consumption	Tanzania

Nwatu et al. 2017 [27]	≥18	34.8%	834	– sex: male – BMI > 25 kg/m^2^ – age > 45 years – physical inactivity – impaired glucose tolerance	Nigeria

Muchanga et al. 2016 [28]	40–60	38.5 %	200	– menopause – use of traditional medicine	Congo

Ezekwesili et al. 2016 [29]	17–79	42.54%	912	Not evaluated	Nigeria

Guwatudde et al. 2015 [30]	≥ 18	36.9%.	3906	– Male gender – age: 18 – 19 years	Uganda

Nkeh-Chungag et al. 2015 [31]	13–17	12.3%	388	Not evaluated	South Africa

Abdissa et al. 2015 [32]	≥ 18	47.3%	2716	Not evaluated	Ethiopia

Ellenga Mbolla et al. 2014 [33]	5–18	20.7%	603	– overweight/obesity – secondary school – migration	Congo

Ale et al. 2014 [34]	26–86	43.56%	101	– higher left ventricular mass – higher left ventricular mass index 1 – higher left ventricular mass index 2	Nigeria

Mehdad Silmane et al. 2013 [35]	11–17	9.6%	167	– overweight/obesity – boy	Morocco

Tayel et al. 2013 [36]	12–18	34%		– overweight/obesity – daily intake of energy, macronutrients, sodium, and potassium – consumption of soft drinks	Egypt

Nuwaha et Musinguzi 2013 [37]	≥18	33.9%	4142	– overweight/obesuty – 40 years and above, – smoking, – consumption of alcohol, not being married, – being male	Uganda

Ujunwa et al. 2013 [38]	10–18	17.3%	2694	– female – BMI – non-obese	Nigeria

Allal-Elasmi et al. 2012 [39]	35–69	Males: 56.8% Females: 43.1%	2712	– age – male gender – obesity – abdominal obesity – smoking	Tunisia


### Topics

Several aspects have been addressed in the selected articles such as prevalence and factors associated with prehypertension.

### Prevalence of prehypertension

The prevalence of prehypertension varied from 2.5% to 58.7%. This variation depended on the age of the population. In children and adolescents the prevalence of prehypertension ranged from 2.5% to 34% according to studies [13,15,17,20,24,25,31,33,35,36,38]. Among adults, it varied from 32.9% to 56.8% depending on the country [14,16,18,21,23,27,28,29,30,32,34,37,39]. Some authors worked on populations whose age was between adolescents and adults [19,22,26]. In these studies, prevalence of prehypertension ranged from 29% to 36.2%. In several studies, the prevalence rate of prehypertension was approximately 2 to 3 times higher than that of hypertension.

### Risk factors

Some factors have been associated with prehypertension or with prehypertension prediction. These factors were: socio demographic caracteristics (age, sex and level of education, place of residence), lifestyle (sedentarity, low physical activity, smoking, alcohol consumption), alimentation (low fruits and vegetables consumption, eating fried food, drinking sugar soft drinks), overweight, obesity, abdominal obesity, cardiometabolic and electrocardiographic characteristics.

#### Children and adolescents

Several risk factors for prehypertension have been identified. These risk factors were dominated by overweight and obesity [13,15,20,24,25,33,36]. Only one author found that being non-obese was linked to prehypertension [38]. In addition to these factors, Redjala et al. observed that more than 2 hours per day spent watching TV, internet and electronic games, parental hypertension or diabetes, shorter gestational age (33 – 36 weeks), early birth, reduced birth weight and shorter breastfeeding were correlated with prehypertension. Regarding age, some authors noted that an age greater than 10 years was a risk factor for prehypertension [15,20]. Concerning gender, Sungwa and Ujunwa [15,38] reported that prehypertension was related to female gender, whereas according to Mehdad [35] male gender was a risk factor for prehypertension. Other factors have been found in children and adolescents such as eating fried food, drinking sugar soft drinks, not eating fruits, daily intake of energy, macronutrients, sodium, and potassium [15,36]; secondary school [33] and public school [24].

#### Adults

**- Socio demographic characteristics (age, sex, level of education and place of residence)**

Some studies showed an association between prehypertension and age [16,19,23,27,37,39]. The association between prehypertension and sex has been demonstrated in various studies [14,19,22,27,30,39]. Almost all of these studies have noted that the risk of developing prehypertension was higher in males than female. Few studies have worked on the educational level and place of residence. Some authors found that having a lower level of education was significantly associated with prehypertension [16,18,26] while two authors noticed that one of the factors associated with prehypertension was place of residence [16,26].

**- Lifestyle and alimentation**

A number of research have noted a link between prehypertension, people’s lifestyles and alimentation [14,16,18,19,26,37,39]. Sedentarity lifestyle, not practicing at least 30 min daily walks, low physical activity, smoking and alcohol consumption were associated with prehypertension [14,18,26,37,39]. Likewise, low fruit and vegetable intake not eating fruits and vegetables were risks factors for prehypertension [14,16,19].

**- Overweight and obesity**

Many surveys have shown that BMI was correlated with prehypertension [14,16,23,26,27,37,39].

Oveweight and obesity [14,16,22,26,27,37]; obesity and abdominal obesity [23,39] were risk factors for prehypertension described by several authors.

**- Cardiometabolic factors**

Some studies have looked for a link between cardiometabolic factors with prehypertension [27,34].

Nwatu et al. [27] have shown that impaired glucose tolerance were significant predictors of prehypertension. Ale et al. [34] measured the impact of prehypertension on some electrocardiographics and echographic factors. They noticed that compared with normotension, prehypertension was associated with higher left ventricular mass and higher left ventricular mass index.

**- Other factors**

Few other factors have been associated with prehypertension Muchanga et al. [28] found that menopause, the use of traditional medicine and haemoglobin levels were associated with prehypertension while Nuwaha [37] reported that being married was a risk factor for prehypertension.

## Discussion

In Africa, some authors have worked on prehypertension, however, to our knowledge, there is not yet a systematic review on this subject. Prevalences have been estimated on the basis of surveys carried out in some localities of the countries concerned. Thus, global or sufficiently representative figures for the prevalence of prehypertension in African countries must be evaluated.

This review allowed us to observe that the prevalence of prehypertension was relatively higher than that of hypertension in the different samples analyzed. This observation suggests that cardiovascular disease prevention policies must take into account prehypertensive populations in particular.

According to the association between socio-demographic characteristics and prehypertension, there was a trend towards a positive link with age and gender. This association was also noticed in other parts of the world [40,41,42]. The association between prehypertension and level of education was poorly documented in this review. However, studies reported that a low educational level in adults was positively associated with a prehypertension.

The association with prehypertension and lifestyle and obesity was well documented in over part of the world [42,43,44]. Regarding alcohol consumption, a systematic review noted that, in people who drank more than two drinks per day, a reduction in alcohol intake was associated with increased blood pressure reduction [45]. Lifestyle such as cigarette smoking increases blood pressure and is an exogenous risk factor for prehypertension and other cardiovascular diseases. This review concurs with recent studies’ findings, which concluded that cigarette smoking damages the arterial wall and increases the blood pressure in adults, resulting in prehypertension. The negative effects of lack of physical activity, overweight and obesity on health and particularly on prehypertension is well documented. Health programmes and policies to promote physical activity and reduce overweight and obesity must be undertaken. This initiative will help to reduce not only prehypertension and cardiovascular diseases but also other non-communicable diseases associated with obesity such as cancers.

## Limitations

We included only PubMed and google scholar in our search and our review was limited to articles published in English and french, which raises the possibility of omissions.
